# Feasibility of visual instrumented movement feedback therapy in individuals with motor incomplete spinal cord injury walking on a treadmill

**DOI:** 10.3389/fnhum.2014.00416

**Published:** 2014-06-12

**Authors:** Daniel Schließmann, Christian Schuld, Matthias Schneiders, Steffen Derlien, Maria Glöckner, Till Gladow, Norbert Weidner, Rüdiger Rupp

**Affiliations:** ^1^Experimental Neurorehabilitation, Spinal Cord Injury Center, Heidelberg University HospitalHeidelberg, Germany; ^2^Institut für Physiotherapie, University Hospital JenaJena, Germany; ^3^HASOMED GmbHMagdeburg, Germany

**Keywords:** spinal cord injury, visual realtime feedback, proprioception, gait rehabilitation, treadmill, motion analysis, motor learning

## Abstract

**Background:** Incomplete spinal cord injury (iSCI) leads to motor and sensory deficits. Even in ambulatory persons with good motor function an impaired proprioception may result in an insecure gait. Limited internal afferent feedback (FB) can be compensated by provision of external FB by therapists or technical systems. Progress in computational power of motion analysis systems allows for implementation of instrumented real-time FB. The aim of this study was to test if individuals with iSCI can normalize their gait kinematics during FB and more importantly maintain an improvement after therapy.

**Methods:** Individuals with chronic iSCI had to complete 6 days (1 day per week) of treadmill-based FB training with a 2 weeks pause after 3 days of training. Each day consists of an initial gait analysis followed by 2 blocks with FB/no-FB. During FB the deviation of the mean knee angle during swing from a speed matched reference (norm distance, ND) is visualized as a number. The task consists of lowering the ND, which was updated after every stride. Prior to the tests in patients the in-house developed FB implementation was tested in healthy subjects with an artificial movement task.

**Results:** Four of five study participants benefited from FB in the short and medium term. Decrease of mean ND was highest during the first 3 sessions (from 3.93 ± 1.54 to 2.18 ± 1.04). After the pause mean ND stayed in the same range than before. In the last 3 sessions the mean ND decreased slower (2.40 ± 1.18 to 2.20 ± 0.90). Direct influences of FB ranged from 60 to 15% of reduction in mean ND compared to initial gait analysis and from 20 to 1% compared to no-FB sessions.

**Conclusions:** Instrumented kinematic real-time FB may serve as an effective adjunct to established gait therapies in normalizing the gait pattern after incomplete spinal cord injury. Further studies with larger patient groups need to prove long term learning and the successful transfer of newly acquired skills to activities of daily living.

## Introduction

A spinal cord injury (SCI) leads to persistent sensorimotor impairments in the lower (paraplegia) and upper (tetraplegia) extremities (McDonald and Sadowsky, 2002) resulting in limitations of mobility and participation in society. Over the last decade a clear trend toward a higher proportion of cases with motor incomplete SCI (iSCI) can be seen worldwide. Currently about 60% of all new spinal cord injuries are incomplete (National Spinal Cord Injury Statistical Center, 2013). Individuals with initially preserved motor functions below the level of lesion have a good prognosis to become ambulatory after 3–6 month (Katoh and el Masry, 1995; Burns et al., 1997). However, even in those subjects who reach a sufficient level of ambulation, sensory and in particular proprioceptive impairments or spasticity may be present and limiting walking function. Restrictions in deep sensibility lead to an altered or absent intrinsic afferent feedback to the central nervous system. It is known that a loss of afferent feedback severely affects motor control particularly within a changing environment (Riemann and Lephart, 2002; Schmidt and Lee, 2011). Therefore, one characteristic of neurological gait disorders is a non-physiological and instable gait pattern, often accompanied by the fear to fall (Sanes et al., 1985; Dietz, 2002). Under normal conditions the control of the walking pattern requires little attention. However, adaptation of the walking pattern to changing environments is mainly controlled by cerebellar and cerebral motor areas (Morton and Bastian, 2006). These conscious processes preferentially access the spatial and not the temporal walking pattern (Malone and Bastian, 2010).

If intrinsic feedback is impaired, extrinsic feedback (FB) provided by human therapists or technical systems may compensate for the loss of sensory function and may help to restore a physiological walking pattern. It was shown that patients with cerebral palsy are able to normalize their pathological gait pattern, if deviations from a normal walking pattern are fed back while walking in a locomotion robot on a treadmill (Labruyere et al., 2013; Meyer-Heim and van Hedel, 2013). Within a training program following motor learning principles FB is an effective tool to boost motor learning (Krakauer, 2006). It can be provided using haptic, visual, auditory modalities either alone or in combinations, because all have dedicated strengths and weaknesses (Sigrist et al., 2011, 2013). Besides modality, FB frequency and timing are important factors for performance and learning efficacy (Park et al., 2000; Maslovat et al., 2009; Sigrist et al., 2013). High-frequency or concurrent FB seems to be beneficial for inexperienced learners and for those confronted with complex tasks (Wulf et al., 1998; Wulf and Shea, 2002), whereas for simple motor tasks lower FB frequencies seem to be more beneficial (Maslovat et al., 2009).

While principles of motor learning and FB are well investigated in sport sciences and commonly applied in optimizing sportsmen performance (Wulf et al., 2002; Anderson et al., 2005; Schmidt and Lee, 2011; Sigrist et al., 2011; Keogh and Hume, 2012; Thow et al., 2012), the integration of FB methods in gait rehabilitation programs of iSCI patients has just recently started (Banz et al., 2008; Duschau-Wicke et al., 2010; Schuck et al., 2012; Govil and Noohu, 2013). Most studies related to FB during walking are based on the Lokomat, which is a motor-driven gait orthosis for robotic assistance during treadmill training (Colombo et al., 2001). Beside its actuator components the device can be extended to use sensor data for quantification of hip and knee joint angles and torques for FB (Duschau-Wicke et al., 2010; Schuck et al., 2012). Due to the small number of sensors important gait parameters e.g., kinematics of the feet cannot be assessed easily. Additionally, information about the natural walking pattern of a user cannot be obtained with the locomotion robot due to its mechanical constraints allowing only movements in the sagittal plane.

Another issue is the implementation of the feedback. In a recent study focusing on the feasibility of patient-cooperative robotic gait training FB was provided in form of a moving reference avatar, which is overlaid by the real kinematic parameter of the user (Schuck et al., 2012). Although, the specific contribution of the FB on the improvement of overground walking were not assessed and systematic investigations are missing, based on clinical experience it can be assumed that this rather unspecific FB may be less efficient compared to joint or segment specific FB. This statement is underlined by the results of a recently published randomized controlled study, which showed positive effects of FB of the electromyogram derived from the M. gluteus maximus on the walking function of individuals with iSCI (Govil and Noohu, 2013). However, in already ambulatory individuals it might be more important to achieve a more physiological walking pattern to avoid long-term complications instead of a higher walking velocity. If a more physiological walking pattern is in the focus of the therapy, a kinematic gait analysis is indispensable.

So far all studies investigating the effect of FB on the walking pattern of individuals with iSCI have shown that (1) motivation rises resulting in a more active participation of the participants during the training sessions (Krakauer, 2006), that (2) they are able to alter their gait pattern during FB and that (3) positive effects on the gait capabilities after the FB training can be maintained in the short-term. However, retention and potential learning effects during no-FB sessions after a longer pause period are mainly unknown. Hence to date, it is not entirely clear if subjects with iSCI and the associated sensorimotor impairments are able to influence their pathological gait pattern during FB application. Most importantly, it is still an open question, if iSCI subjects have an improved long-term outcome i.e., that the application of FB has the characteristics of a therapy. The following study represents a feasibility study aiming at improving the gait pattern of ambulatory chronic iSCI individuals with predominantly sensory impairments by application of a novel motion analysis based real-time FB system. The technical implementation of the feedback modality had to be tested with motor unimpaired subjects in a pilot study before applying it to individuals with iSCI.

## Materials and methods

### Motion analysis and feedback system

**Figure 1:**
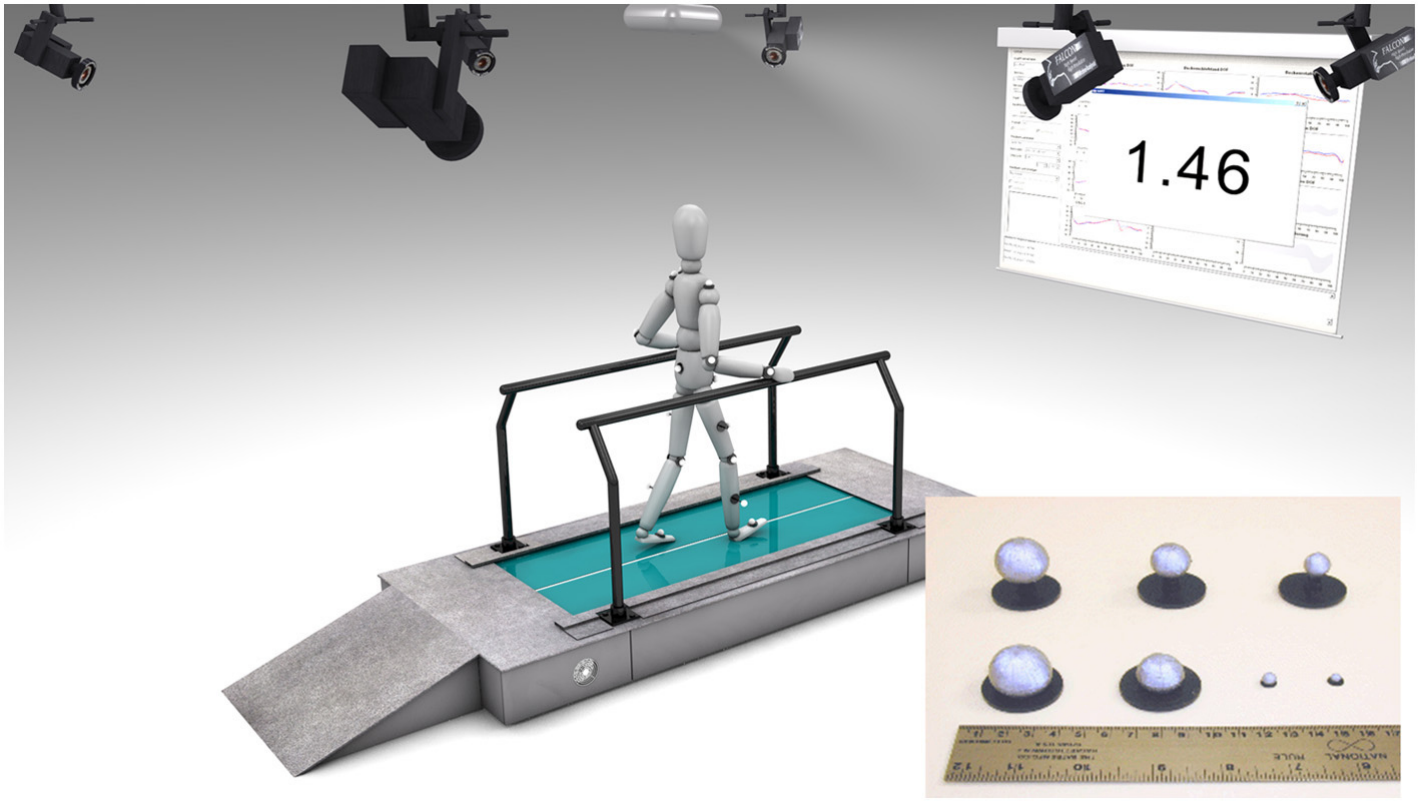
**Components of the feedback system used in this study**. Infrared motion capture system using reflective markers associated with a customized treadmill. Knee joint angles are calculated in real-time during every step and compared to reference values. The calculated norm distance value is fed back to the participant using a projector and canvas.

For the intended study a dedicated FB system featuring real-time motion analysis (Motion Analysis Inc., Santa Rosa, CA, USA) consisting of 8 Hawk cameras to capture a volume of approximately 1.5 × 1.4 × 2 m on a custom-made treadmill (Rupp et al., 1998) and a proprietary FB software (Figure 1) was developed. A standard Helen Hayes marker set (Kadaba et al., 1990) consisting of 20 retroflective markers (Ø 10 mm) together with the dedicated biomechanical model of the lower extremities was used to calculate angles of the hip, knee and ankle joints online with a sampling frequency of 200 Hz. Gait events were estimated algorithmically by detecting local extremes in the trajectories of heel and toe markers by a validated proprietary implementation optimized for low detection latencies (Schablowski-Trautmann, 2005).

The commercial software of the motion analysis system (Cortex) streams marker trajectories, analog data and the model based joint angles with a low latency of less than 2 frames via multicast into the local area network. The low-level module of the proprietary FB software written in C++ using Cortex SDK 4.1.6 (Motion Analysis Inc., Santa Rosa, CA, USA) and the QT toolkit 4.8.4 (Digia Plc, Helsinki, Finland) receives the multicast stream, performs basic signal processing tasks e.g., low latency gait event detection, time distance parameter calculation and gait cycle normalization. The high-level module based on the MATLAB 2010a engine (The MathWorks, Inc., Natick, MA, USA) computes parameters for use as FB values e.g., deviations of the current step from a physiological kinematic walking pattern (see below) or deviations of the focal step from an artificial movement task, like the one used for feasibility testing with healthy individuals (see Pilot study with non-impaired individuals). By making extensive use of high-level MATLAB functions FB parameters can be visualized by a projector on a canvas in 3.5 m distance. FB was provided with a latency of <150 ms after the pre-defined gait event, which is in this case the heel strike.

For implementation of the FB, joint angles were normalized to 100% gait cycle. A scalable norm distance (ND) measure (Wolf et al., 2006) was used as FB parameter in the iSCI cohort. In general, ND reflects the deviations from a physiological gait pattern. It is defined as the difference between the actual, pathological joint angle x_p_ and the mean norm angle *x_n_* of a gait velocity dependent cohort of non-impaired subjects (Schablowski-Trautmann, 2005) weighted by the standard deviation of the norm angle σ_*n*_ at point *k* within a step normalized (0 – 100 percent gait cycle) joint angle:

(1) $$ND_{p}\left[k\right]=\left|\frac{x_{p}\left[k\right]-\bar{x_{n}}\left[k\right]}{\sigma_{n}\left[k\right]}\right|$$

The ND has in contrast to other methods like averaging the inherent advantage of including a-priori knowledge of normal walking when used as a FB parameter. ND values between 0 and 1 indicate physiological gait patterns, whereas values >>1 indicate pathological ones (Schablowski-Trautmann et al., 2006).

### Pilot study with non-impaired individuals

**Figure 2:**
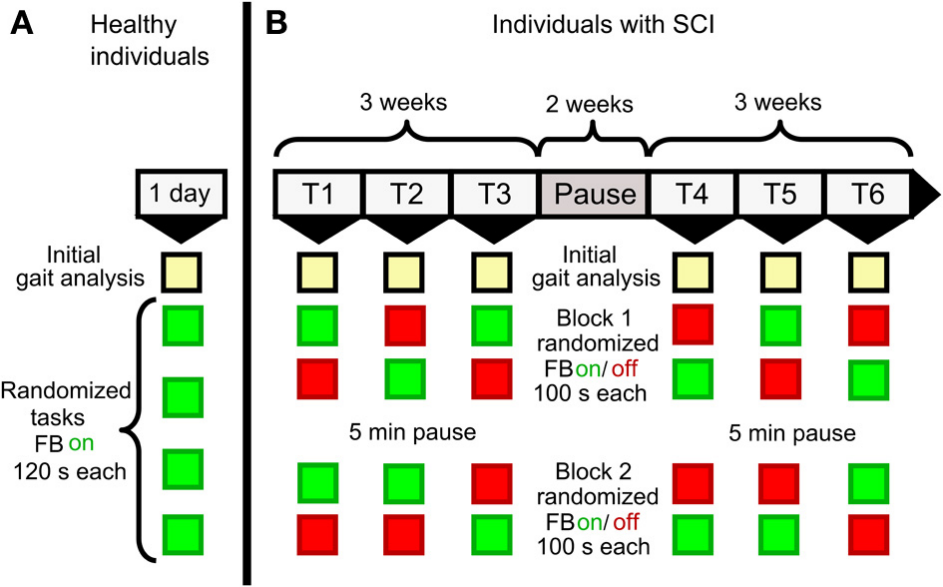
**Study protocol for (A) non-impaired and (B) iSCI individuals**. Non-impaired individuals completed a reference measurement (initial gait analysis) followed by 4 sessions with artificial movement tasks (+20, +40, −20, −40% of previously recorded mean knee angles during normal walking) in randomized order. Individuals with iSCI completed 6 weeks (1 training day per week, two blocks of feedback/ no-feedback sessions per day of training) of FB training with a pause after the third week. Those blocks were randomized, except for the first block of the first day of training, which started with a FB session. Equivalent to the condition in the non-impaired participants each session started with an initial gait analysis.

In a case series motor unimpaired individuals walking on a treadmill received visual FB about their mean knee flexion angle (0° flexion angle means fully extend knee) during swing phase. Treadmill speed was set at 0.8 m/s for all participants. Each participant first started with an initial reference measurement of 120 s to assess the normal walking pattern without FB. This reference measurement was followed by four sessions each lasting for 120 s, in which the subject had to perform different artificial movement tasks with the support of visual FB (Figure 2A). During each session the individuals had to adjust their flexion angle of one knee during swing phase to a predefined percentage (+20, −20, +40, −40%) of the previously determined reference angle, which is the mean knee angle during swing phase averaged over all detected strides on one side. The order of movement tasks, as well as the body side to which FB was applied, was randomized. The definition of the artificial movement tasks on the basis of each individual's own reference angle was made to assure that tasks were equally challenging for each participant.

**Figure 3:**
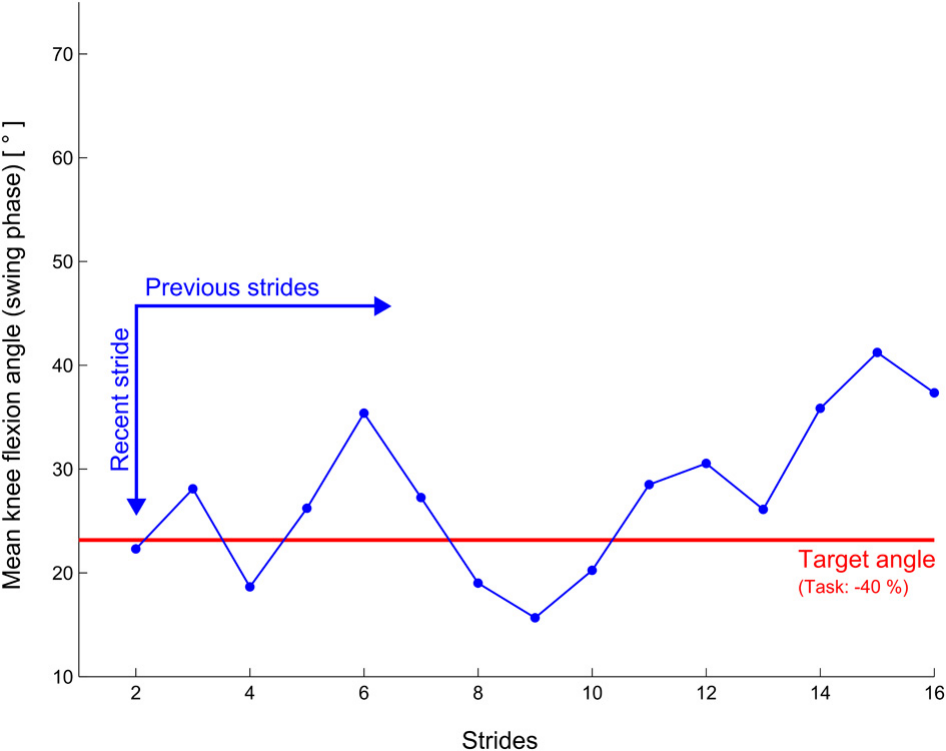
**Illustration of the feedback display of the pilot study with non-impaired subjects**. The blue graph is shifted to the right on every stride.

Visual FB consisted of a line diagram (Figure 3) where the mean knee flexion angle during swing phase is displayed on the y-axis and the latest 16 FB values on the x-axis. The diagram was updated after every stride of the respective side. A red horizontal line indicated the optimum of the movement task i.e., the desired mean knee flexion angle during swing phase. After receiving a brief verbal introduction on relevant gait events, the movement tasks and FB implementation, the participants were instructed to adjust their knee flexion in order to match the FB value as good as possible to the red line. The different movement tasks were presented consecutively with about 10 s pause in between to let the operator switch to the next task. Participants were asked to walk normally during this transition period.

A movement task was considered successfully performed, if the stepwise mean group performance accuracy as measured by the accumulated standard error dropped below 1°.

### Feasibility study with iSCI individuals

For the feasibility study, individuals with chronic iSCI (date of injury >12 months) and predominantly sensory deficits in the lower extremities were included. Additionally, basic walking function i.e., walking with a walker or with less support by walking aids, was preserved (Walking Index for Spinal Cord Injury II (Marino et al., 2010) (WISCI II) ≥13). Patients were recruited by screening of the medical records of the SCI Center of the Heidelberg University Hospital. Subjects with stiff-knee gait pattern were selected by observational gait analysis. A stiff-knee gait is characterized by an insufficient flexion of the knee during swing phase. Hand and arm function had to be preserved to a degree sufficient to hold on to parallel bars during treadmill walking.

Each individual completed 2 episodes of 3 weeks of FB training on the treadmill with 1 training day per week and a 2 weeks pause after the first episode (Figure 2B) resulting in 6 training days in total. Every training day started with an initial gait analysis to assess the individual's current gait kinematics followed by 2 therapy blocks each consisting of one FB/no-FB session (100 s each) in randomized order, except for the very first block on the first training day, which always started with a FB session. To follow the idea of distributed practice (Krakauer, 2006) therapy blocks included no-FB sessions to avoid full dependence on FB but rather promote medium and long-term motor learning. Visual FB was given after every stride as an absolute number of the ND (see equation 1) of the knee angle during swing phase of the most affected leg. The number was updated on every heel strike of this leg. Font size was set to 180 points to ensure legibility for every participant and the number was displayed with two fractional digits (Figure 1). In contrast to the pilot study with non-impaired individuals, only numerical and no graphical representation of ND was presented. Prior to FB sessions, individuals were instructed to actively lower this number by altering their gait pattern. The operator suggested movement strategies such as “Perform faster thigh movements during hip flexion” or “Actively increase your knee flexion by trying to lift the heel up closer to the buttock.” Additionally, hints for altering cadence and foot placement were given that might help an individual to achieve a more physiological gait pattern. The verbal instructions were the same for FB and no-FB sessions, where individuals were asked to focus on the movement task. During walking sessions the operator reduced communication to a minimum in order to avoid any distraction of the subject from the movement task. For each initial gait analysis, individuals were asked to walk like in daily life, without concentration on their walking pattern. In this case, no additional verbal information was provided. At study inclusion, treadmill speed was adjusted to the individual comfortable walking speed to minimize mental and physical fatigue during the training session. The self-selected walking speed of each individual was kept constant during all sessions. Keeping the treadmill speed constant is a prerequisite for comparing the NDs between sessions due to the large influence of walking speed on gait kinematics (Schablowski-Trautmann et al., 2006).

For standardized assessment of the SCI related sensorimotor deficits motor and sensory (pin prick discrimination, light touch appreciation) examinations were performed at study onset according to the International Standards for Neurological Classification of Spinal Cord Injury (ISNCSCI) published by the American Spinal Injury Association (ASIA) (Kirshblum et al., 2011). A high-quality ISNCSCI assessment was assured by trained assessors (Schuld et al., 2013) and computational scaling, scoring and classification (Schuld et al., 2012) including the ASIA Impairment Scale. The preserved degree of proprioception in the knee joint was assessed prior to the first training session by a vibratory sensation testing at the epicondylus medialis femoris of the leg selected for FB using a tuning fork (64 Hz testing frequency, scale 0–8, minimal to maximal sensitivity).

Confirmatory statistics were not considered due to the small sample size and the feasibility character of the study. Therefore, only descriptive statistics were performed. The main goal of the study was to test the feasibility of this completely new therapy approach in iSCI and to determine effect sizes for future clinical trials. Descriptive statistics and visualization were performed using R 3.0.0 (R Core Team, 2012) and MATLAB 2010a. This feasibility study has been approved by the institutional review board. All non-impaired and impaired study participants gave written informed consent.

## Results

### Pilot study

**Figure 4:**
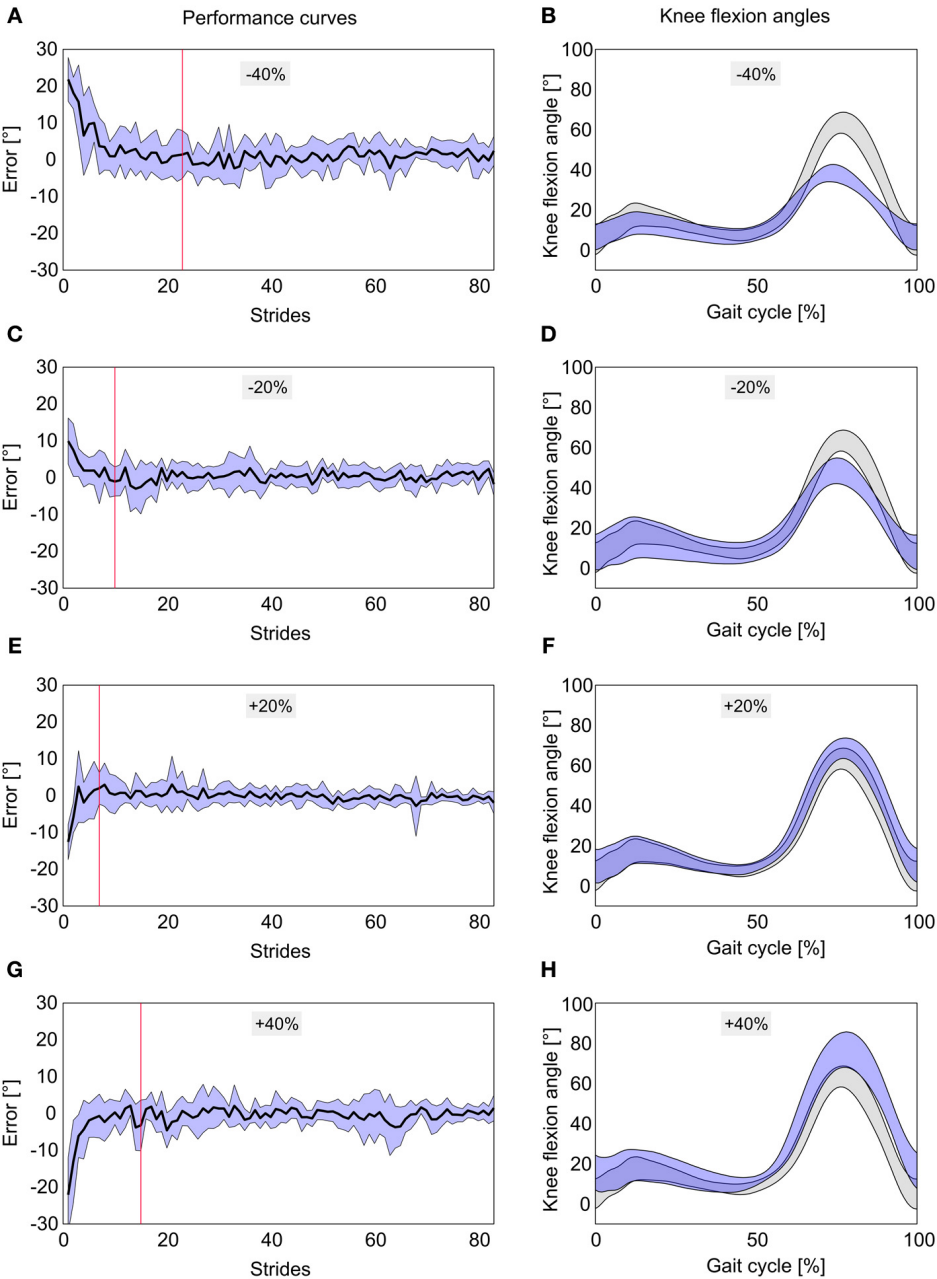
**Performance curves and mean knee flexion angles to the movement tasks −40% (A,B), −20% (C,D), +20% (E,F), and +40% (G,H) of normal knee flexion angles**. Performance curves display 83 steps during feedback training averaged over 9 non-impaired individuals ± *SD* (blue area). Red vertical lines mark the point where the SE of the mean error is within ±1°. Mean knee flexion angles are displayed as blue curves averaged over 9 non-impaired individuals. Gray curves represent mean knee flexion angles ± *SD* of all participants during normal walking (initial gait analysis).

Nine motor unimpaired individuals (age: 31.4 ± 5.3 years; 2 females, 7 males, Table 1) completed the FB training. All participants managed to perform every movement task within the training duration of 120 s. Figure 4 depicts the mean performance curves per task. The number of strides varied within participants and tasks. Accordingly, the ensemble average was only calculated for the least common number of strides per person and session, which was 83 for this study.

**Table 1:** **Data of non-impaired individuals participating in the pilot study**.

**ID**	**Sex**	**FB side**	**Age (years)**	**Mean swing knee flexion angle [°]**
10569	m	R	35	48.01
10672	f	L	23	38.06
10671	f	R	28	32.28
10440	m	L	38	36.34
10144	m	L	38	46.45
10635	m	R	34	43.46
10588	m	R	26	40.74
10567	m	R	31	41.80
10620	m	L	30	48.66
		Mean	31.44	41.76
		*SD*	5.25	5.54

Mean knee flexion angle during swing phase as recorded in the reference measurement episode.

The −40% task was successfully performed after 23 strides (red line in Figure 4A) and the mean error during the FB trial was 1.87 ± 3.88°. During the movement task of −20% the desired knee flexion angle was reached after 10 strides (Figure 4C) with a mean error of 0.58 ± 1.85°. The +20% task was successfully fulfilled after 7 strides with a mean error of −0.30 ± 1.85^°^ (Figure 4E) and +40% after 15 strides with a mean error of −0.92 ± 3.17^°^ (Figure 4G) respectively. Movement tasks with positive algebraic signs which needed an adjustment of the mean knee angle during swing phase toward larger values yielded slightly smaller outcome values both in movement task approximation and mean errors compared to their negative counterparts. Reflecting the magnitude of the before mentioned average mean errors of the gait tasks, gait cycle curves were below (Figures 4B,D) and slightly above (Figures 4F,H) the mean normal gait cycle during swing phase.

### Feasibility study

**Figure 5:**
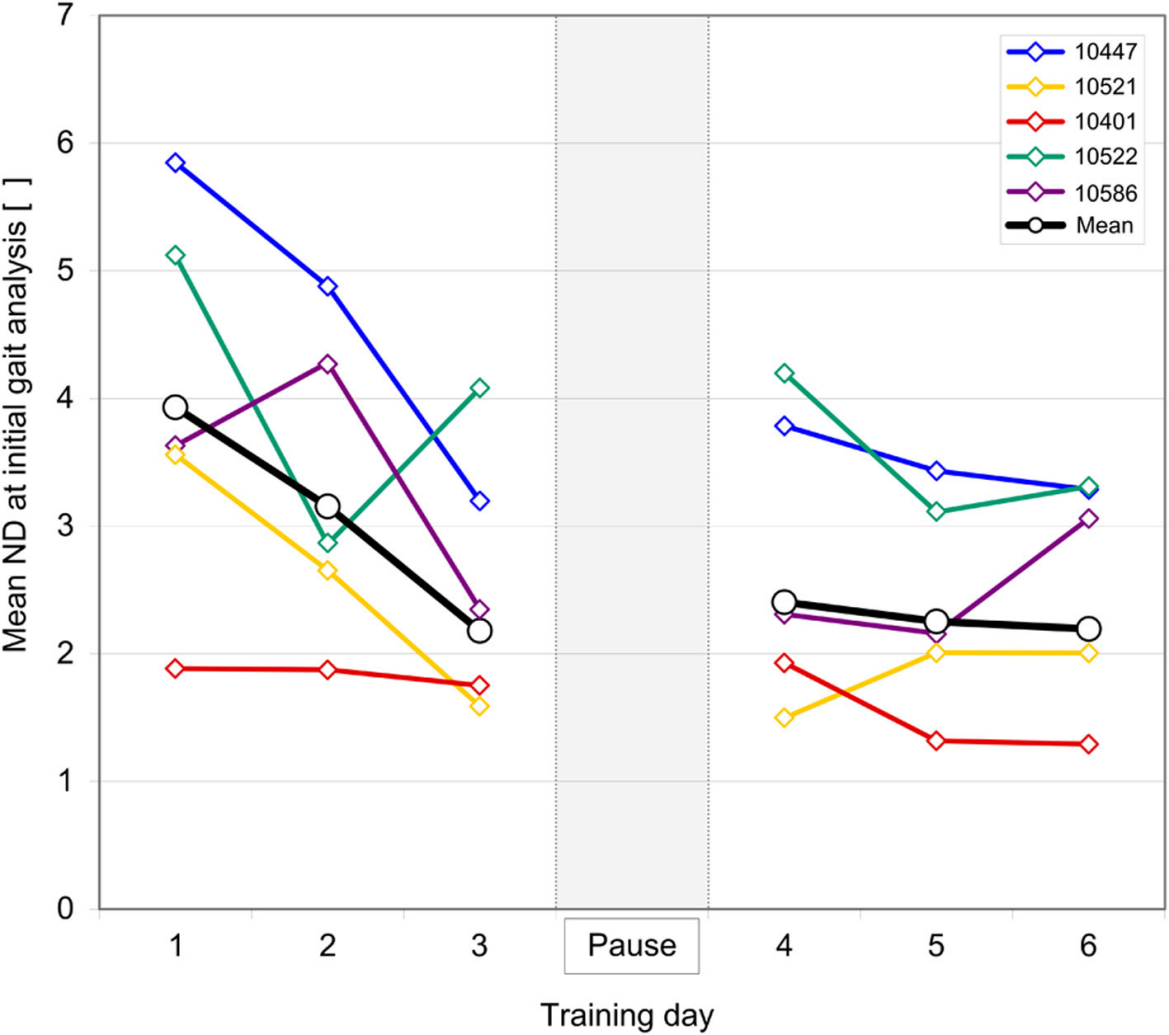
**Learning curves of individuals with iSCI over 6 days of training with a 2 weeks pause in between**. Norm distance (ND) values were derived from initial gait analysis (without feedback) preceding every feedback session. Brown lines indicate mean ND over all individuals with iSCI.

**Figure 6:**
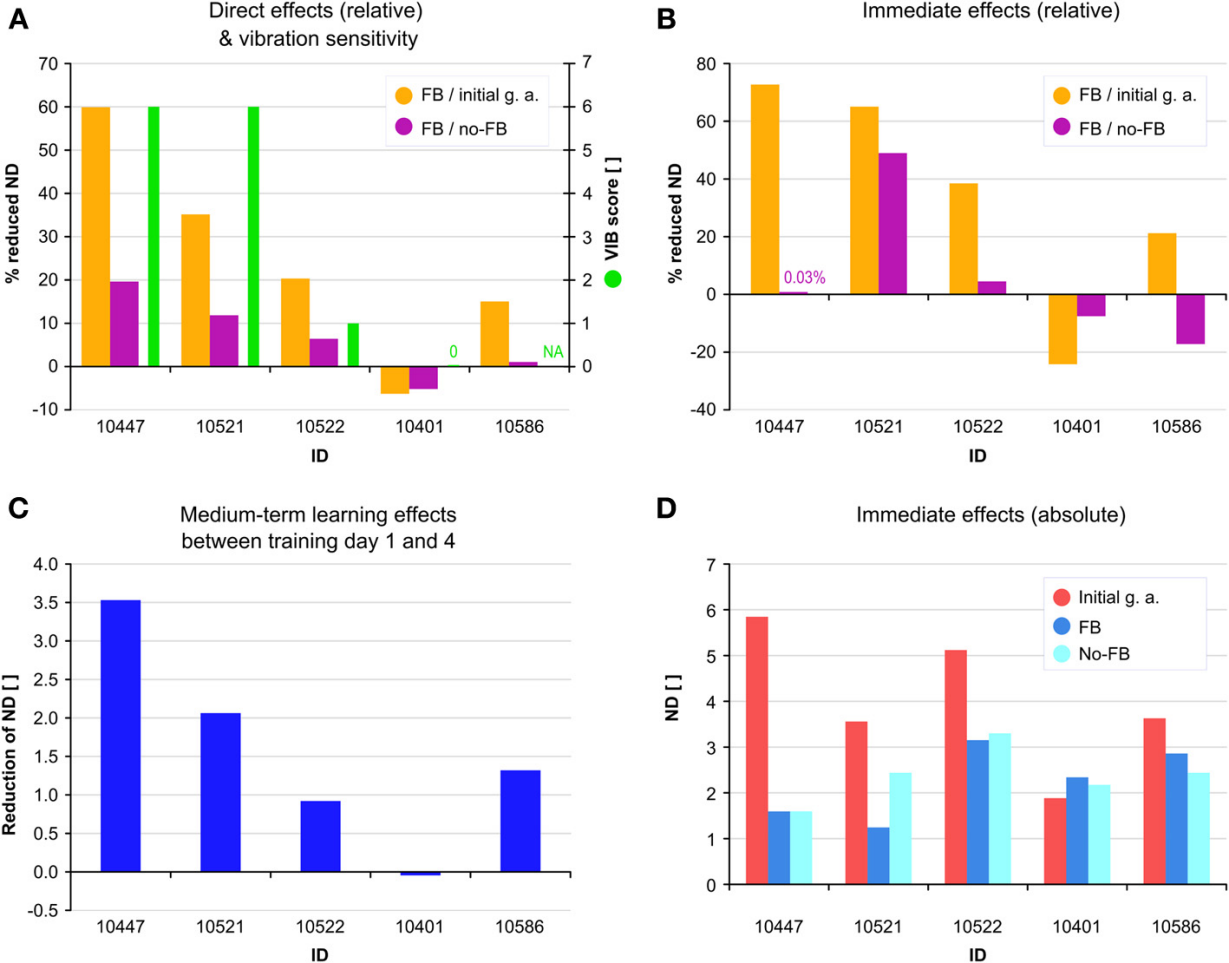
**(A)** Direct effects of feedback FB as reduced norm distance (ND) during all FB sessions relative to all initial gait analyses (orange) and relative to all no-FB sessions (purple). Vibration sensitivity (VIB) score is displayed as green bars. **(B)** Immediate effects of FB during the first FB session relative to the first initial gait analysis (orange) and relative to the first no-FB session (purple). **(C)** Medium-term learning effects as reduction of ND between day 1 and 4 of training. **(D)** Immediate effects of FB displayed as absolute values in ND during the first initial gait analysis (red), first FB session (blue) and first no-FB session (bright blue).

Five chronic (23.6 ± 8.6 months after injury) individuals (age: 55.2 ± 8.5 year, 3 female, 2 male) with iSCI (4 ASIA Impairment Scale (AIS) D, 1 AIS B) successfully completed all 6 days of training (self-selected gait speed 0.5 ± 0.1) (Table 2). Individuals with iSCI showed a strong decrease in mean ND of the initial gait analysis without FB from 3.93 ± 1.54 on the first day of training to 2.20 ± 0.90 on the sixth day of training. The strongest decrease of 1.75 (3.93 ± 1.54 to 2.18 ± 1.04) is observed over the course of the first three sessions (Figure 5). After a 2 week pause, on the fourth day of training mean ND remains on the same level compared to the third day (2.18 ± 1.04 and 2.40 ± 1.18, respectively) before the pause. Compared to the first half of the total days of training a smaller decrease of 0.21 (2.40 ± 1.18 to 2.20 ± 0.90) occurred over the second half. Considerable direct benefits of FB occurred in four individuals with a significant decrease of the mean ND of all FB sessions (ranging from 60 to 15%) relative to all initial gait analyses. Direct benefits of FB sessions relative to no-FB sessions ranged from 20 to 1% (Figure 6A). Individual 10401 showed a slight decrease in performance (−6 and −5%, respectively). Four individuals reduced their ND already in the first FB session (Figure 6B). The decrease in ND observed in the first FB sessions ranged from 73 to 21% relative to the first initial gait analysis, and −24% for individual 10401. Decreases in ND of the first FB session relative to the first no-FB session ranged from 49 to 0.03%. Individuals 10401 and 10586 showed increases in ND (−7.5 and −17%, respectively).

**Table 2:** **Characteristics of individuals with SCI included in the feasibility study**.

**ID**	**Sex**	**AIS**	**NLI**	**Age**	**MAI**	**LEMS (max. 50)**	**PP (max. 112)**	**LT (max. 112)**	**VIB (max. 8)**	**WISCI II (max. 20)**	**S [m/s]**
10447	m	D	Th8	42	22	47	73	88	6	16	0.6
10521	f	B	L3	65	25	47	96	102	6	13	0.6
10522	f	D	C6	59	23	48	109	108	1	20	0.46
10401	f	D	Th6	54	36	43	107	91	0	16	0.42
10586	m	D	Th10	56	12	48	93	107	NA	16	0.4
			Mean	55.2	23.6	46.6	95.6	99.2	3.25	16.2	0.5
			*SD*	8.5	8.6	2.1	14.4	9.2	3.2	2.5	0.1

*Age, sex, months after injury (MAI), data according to the International Standards for Neurological Classification of Spinal Cord Injury (ISNCSCI): Asia Impairment Scale (AIS), Neurological Level of Injury (NLI), Lower Extremity Motor Score (LEMS), Pin Prick score (PP), Light Touch score (LT), and the Walking Index for Spinal Cord Injury II (WISCI II). Knee joint vibration sensation was measured (VIB) as an estimation for proprioception. Treadmill speed (S) was set to the physical capability of each individual*.

Vibration sensitivity in the knee joints was measured for all individuals, however due to hyperaesthesia, individual 10,586 did not yield any reliable data for this assessment. Individuals with a high sensitivity to vibration in the knee joint showed a high immediate benefit from FB training—both related to initial and no-FB trails—, whereas in patients with lower or no sensitivity lower direct and immediate benefits from FB were observed (Figures 6A,B). This correlation can also be seen at a medium-term learning level, displayed as the individual difference in ND between the first initial gait analysis and the first gait analysis after a 2 weeks pause (Figure 6C). Individuals with a high immediate benefit from FB also showed high levels of retention after the 2 weeks pause (Figures 6B,C).

**Figure 7:**
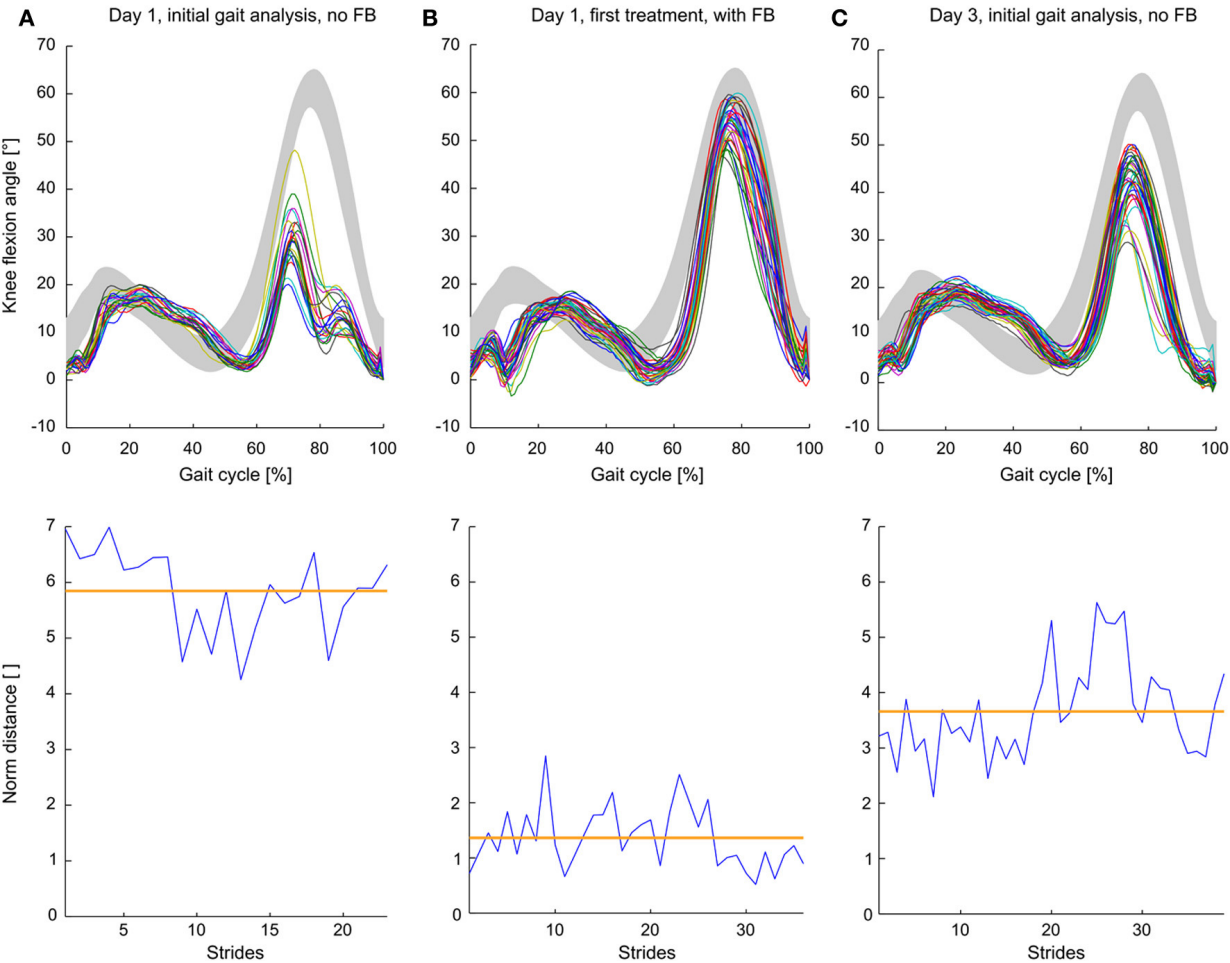
**Step normalized knee angle curves and norm distance values of a single individual with iSCI during (A) initial gait analysis (no feedback) on day 1, (B) first feedback trial during the first day of training and (C) initial gait analysis (no feedback) on the third day**. Gray curves depict mean knee flexion angles ± *SD* of non-impaired subjects. Orange lines show mean norm distance values.

To visualize the relation between ND and kinematics and the effect of FB on the knee angle trajectory, short (within session) and medium-term benefits are exemplarily described for individual 10447 (Figure 7). The stiff-knee gait with decreased knee flexion during swing phase has been confirmed by the initial gait analysis (Figure 7A) with a high mean ND value of 5.85. During the FB trial, swing phase kinematics approached the norm trajectory and ND values decreased to 1.36 (Figure 7B). The initial gait analysis of the third training session showed an intermediate ND value of 3.66 and a knee flexion angle with an intermediate distance to the norm curve (Figure 7C).

## Discussion

### FB in non-impaired subjects

Our results show that the implemented visual FB setup of frequent terminal FB using a novel treadmill based real-time FB system is effective in supporting non-impaired individuals to alter their normal gait patterns. The artificial movement task of precisely altering the knee flexion angle during swing phase could be mastered with support of FB. In our setup the participants successfully managed to adapt their walking pattern to the movement task within a few steps, which represents a very short adaptation time. In another study qualitative real-time video feedback was used to support study participants in improving their gait symmetry on a split-belt treadmill (Malone and Bastian, 2010). The study results show that it took healthy subject approximately 250 strides to compensate for the gait deviations induced by the different belt speeds. Our findings either lead to the conclusions that the tasks were very easy, or that the implemented FB paradigm was effective, or both. Most volunteers judged the task as easy and the visual display as comprehensive. However, lacking any retention test we only assessed performance but not motor learning. Interestingly, tasks requiring less knee flexion during swing phase i.e., −20 and −40%, appeared to be slightly more difficult than the ones requiring more knee flexion. One possible explanation could be that increased knee flexion is commonly used for obstacle stepping in daily life and thus inherent to each participant's movement repertoire.

In conclusion, this experiment shows the technical feasibility of the FB system and confirms our hypothesis that the modulation of knee angles during swing phase is an easy task for healthy individuals, which is a mandatory prerequisite for providing this type of FB for iSCI subjects.

### FB in individuals with iSCI

This study is to our knowledge the first 3 dimensional lower-body kinematic approach for a real-time FB therapy in individuals with iSCI. Our results showed that 4 out of 5 chronic iSCI individuals with stiff-knee gait and predominant sensory impairments are capable to adapt their pathological gait pattern toward more physiological knee kinematics during the application of visual FB. More importantly, the carry-over effects seen after 2 weeks of pause confirm that at least medium-term motor learning occurred. Study participants continued their therapy program during the study. Therefore, the improvements occurred during the study cannot be attributed to changes in the regular training intensity, which qualifies the application of real-time FB as a therapy in individuals with iSCI.

Beyond the expected benefits of FB in the motor learning context, it can be assumed that discussing the results of the initial gait analysis with the patients supported participants in consciously altering their otherwise unconsciously imprinted pathologic motor programs. This is indicated by the strong immediate effects between initial gait analysis and first FB session on the first training day. The extent and direction of this immediate change (Figures 6B,D) seems to reflect the size of the medium-term (Figure 6C) and the direct effects (Figure 6A). Contrary, when FB was switched off during the first no-FB trial (Figure 6B), patients showed very diverse short-term carry-over effects ranging from unchanged performance over considerable performance loss up to performance gains (individual 10,586). Of course, these observations are made on an individual level during the very first application of the FB system and may therefore not be representative for the progress of each individual over the course of the study. Similar to healthy subjects individuals with iSCI can adapt their kinematic pattern to the given movement task within the first FB session. As the amount of individual improvement within the first FB session seems to be associated with direct- and medium-term effects, the improvement during the initial session may identify therapy responders and serve as a marker for the final therapy outcome. In addition to the previously discussed conscious altering of movement patterns, some form of learning could also have occurred on the spinal level (Wolpaw and Carp, 1990; Thompson and Wolpaw, 2014). Due to the fact that already in the first session, which lasts for 200 s, positive effects of FB training occurred, we suggest that alterations in conscious supraspinal motor control most contribute to the early changes in motor behavior and task performance. On the medium- and long-term, adaptations on spinal level might contribute to the manifestation of the more physiological movement pattern.

The individuals with iSCI differed substantially in vibration sensing in the knee joint and in initial performance regarding ND of their treated leg. Interestingly, we observed that patients with low vibration sensitivity benefited less from FB directly, as well as in the medium-term. This observation does not support the assumption that FB may totally compensate for sensory impairments. For the adaptation of the kinematic pattern during FB sessions and for the formation or alteration of a motor program afferent, proprioceptive information is needed (Nielsen and Sinkjaer, 2002; Schmidt and Wrisberg, 2008). Our study participants only received FB in the form of knowledge of results through a rather abstract number after the stride. According to the specificity of learning hypothesis, the most optimal afferent information is integrated into the developing motor program (Proteau, 1992). Considering this, the external FB provided in our specific setup may not be optimal to totally compensate the disturbed internal, proprioceptive feedback. To the contrary, due to its abstract nature, the FB might have provided only moderate guidance (Maslovat et al., 2009; Sigrist et al., 2013), but was intended to promote retention, as individuals were intended to make better use of their preserved proprioception. Our therapy regime represents a motor learning and restorative approach rather than compensatory strategy and therefore does not impose maximal guidance. However, considering the very low sample size, such interpretations must be treated very carefully.

Open questions for future studies include the detection of the right target group for this kind of FB training. Which degree of motor capabilities, proprioception and range of motion are necessary and which degree of spasticity can be tolerated to still achieve a positive therapy effect? Our study participants had an almost unimpaired motor function (LEMS near 50), were able to walk for at least a few minutes and had some spared proprioception at least in the most affected joints. According to our study results, it may be concluded that real-time FB can be effectively used as a therapy in ambulating iSCI patients with predominantly sensory problems.

The effects of FB on gait rehabilitation have been rarely investigated in the iSCI population. Mainly robotic devices have been used (Banz et al., 2008; Schuck et al., 2012) for that purpose. Obvious advantages include prolonged therapy sessions and the potential to include non-ambulating individuals, but the fixed trajectories in the sagittal plane prevent an assessment of natural gait kinematics. With optical motion tracking patients' unrestricted walking pattern can be assessed. This provides patients the freedom of variability e.g., to experiment and solve the movement task in various ways.

On the other hand this freedom in task execution carries some inherent risks, because the movement task can be successfully fulfilled with different strategies. Although the reduction of ND indicates a positive change in the knee kinematics, it does not necessarily indicate a convergence to a more physiological walking pattern of all joints of the lower extremities. As the kinematics of only one joint was in the focus of the FB therapy, the rest of joints could have slinked away from their physiological movement patterns. Therefore, in future studies FB training should be implemented in such a way that the kinematics of the most affected joint should be normalized with the constraint, that kinematics of other ipsilateral and contralateral joints, are kept in a physiological range. Those studies should include proper retention tests directly after end of training and also several months later. FB displays should be more motivating, following a multimodal approach (Sigrist et al., 2013). In analogy to the assist-as-needed principle recently applied in locomotion robots (Emken et al., 2007), FB therapy should accommodate to the patients' progress and needs. This can be done by adjusting FB complexity and frequency and herewith find a compromise between challenging and supporting the patient.

Treadmill walking differs from walking over ground due to the constant velocity, the inability to walk curves, a different optical flow (Brennan et al., 2012) and hand rails for balance and/or body weight support. Additionally, spatial and temporal gait parameters acquired on the treadmill may be transferred differentially to over ground walking (Yen et al., 2012). Therefore, it needs to be investigated, if improvements of our FB training on the treadmill can be transferred to over ground walking. Even though it has been shown that removing vision during treadmill adaptation could improve overground transfer of the new walking pattern (Torres-Oviedo and Bastian, 2010), the ultimate goal would be to train in everyday life situations. For this purpose, mobile gait analysis systems are promising tools that provide the possibility for measurement of joint angles and time-distance parameters. In an ongoing project called “RehaGait” (BMWi, German Federal Ministry of Economic Affairs and Energy, Project number KF2906702KJ2) the commercially available gait analysis system RehaWatch® (HASOMED GmbH, Magdeburg, Germany) based on inertial sensors is currently redesigned to provide FB for different gait affecting conditions (Parkinson's disease, stroke, cerebral palsy, iSCI). Moreover, a portable FB system has enormous spatial and economical advantages compared with a stationary motion-analysis based system, and can address a wide range of patients and institutions. FB in mobile systems can be provided through Augmented Reality glasses, auditory or haptic feedback. This introduces a dual-task in the training, which might degrade the time needed for adaptation, but enhances retention of the newly acquired skills (Torres-Oviedo et al., 2011).

Considering the interesting correlation of the capacity to respond to FB training and vibratory perception, reliable proprioception assessments are desired beyond the discrimination capacity of the tuning fork, which is frequently used in clinical practice as diagnostic tools for polyneuropathy in diabetes (Pourhamidi et al., 2014).

## Conclusion

We show that instrumented real-time movement feedback based on kinematic variables is a promising technique to evoke short- and medium-term changes in individuals with incomplete spinal cord injury and prominent sensory deficits. It can be assumed that FB supported participants in consciously altering their otherwise unconsciously imprinted pathologic motor programs. Further studies including more patients and other gait pathologies are needed to reveal the underlying physiological mechanisms for this observation and to identify the most effective FB parameters, training strategies and characteristics of potential responders. Particularly, the role of proprioception in motor learning of individuals with iSCI needs to be further investigated.

### Conflict of interest statement

The authors declare that the research was conducted in the absence of any commercial or financial relationships that could be construed as a potential conflict of interest.
